# Elucidating the role of Lkb1 and mTOR in adipose tissue

**DOI:** 10.1080/21623945.2018.1535743

**Published:** 2018-10-20

**Authors:** Ziye Xu, Wenjing You, Fengqin Wang, Yizhen Wang, Tizhong Shan

**Affiliations:** a Institute of Feed Science, College of Animal Sciences, Zhejiang University, Hangzhou, Zhejiang, China; b The Key Laboratory of Molecular Animal Nutrition, Ministry of Education, Hangzhou, Zhejiang, China; c Zhejiang Provincial Laboratory of Feed and Animal Nutrition, Zhejiang University, Hangzhou, Zhejiang, China

**Keywords:** adipose tissue, browning, insulin resistance, Lkb1, mTOR, obesity

## Abstract

Adipose tissues, function as energy metabolism and endocrine organ, are closely associated with metabolic diseases such as obesity, insulin resistance and diabetes. Liver kinase B1 (Lkb1) and mechanistic target of rapamycin (mTOR) play crucial roles in regulating energy metabolism and cell growth in adipose tissue. Our recent study generated an adipocyte-specific Lkb1 and mTOR double knockout (DKO) mouse model and found that DKO of Lkb1 and mTOR caused reduction of brown adipose tissue (BAT) and inguinal white adipose tissue (iWAT) mass but increase of liver mass. Moreover, the DKO mice developed fatty liver and insulin resistance but displayed improved glucose tolerance and were resistant to high-fat diet (HFD) -induced obesity. In this commentary, we compare the similarities and differences of the phenotypes found in the DKO mice and Lkb1 or mTOR or mTOR complex 1 (mTORC1) or mTOR complex 2 (mTORC2) single knockout mice. Furthermore, we discuss the potential regulatory mechanism that results in the overlapping or distinct phenotypes found in these models.

Obesity is a worldwide epidemic and serves as a key causal factor of metabolic diseases including insulin resistance, type 2 diabetes, cardiovascular disease, and cancer. Adipose tissue not only plays an important role in regulating energy homeostasis, but also serves as a major nutrient-sensing and endocrine organ regulating whole-body metabolic ﬁtness ^1^. Three types of adipocytes including white, brown and beige adipocytes were found in mammals. White adipocyte stores energy and its expansion results in obesity and insulin resistance. Brown and beige adipocytes, with high levels of uncoupling protein 1 (UCP1) expression, could burn lipid to heat and combat obesity ^2^. Thus, understanding the complicated interplay of the key regulators and biological mechanisms for regulation of the development of white, beige and brown adipocytes is critical and useful for controlling obesity as well as the other metabolic diseases.

Liver kinase B1 (Lkb1) and mechanistic target of rapamycin (mTOR) are key regulators of energy metabolism and cell growth in adipose tissue ^3,4^. In our recent publication, “Adipocyte-specific double knockout of Lkb1 and mTOR protects mice against high-fat diet induced obesity but results in insulin resistance”, published in Journal of Lipid Research, we generated adipocyte-specific Lkb1 and mTOR double knockout (DKO) mice and assessed how genetic knockout of both Lkb1 and mTOR affects adipose tissue development and function in energy homeostasis ^5^. We found that DKO of Lkb1 and mTOR reduced BAT and inguinal WAT (iWAT) mass but increased liver mass ^5^. The DKO mice displayed improved glucose tolerance after high-fat diet (HFD)-feeding and were protected from HFD-induced obesity ^5^. And DKO of Lkb1 and mTOR in adipocytes results in overlapping and distinct metabolic phenotypes compared to Lkb1 or mTOR single knockout ^5^.

mTOR is a well-known engine of anabolic metabolism and poised to be a critical intracellular regulator of adipocyte metabolism, as it acts as a kinase through linking nutrient and hormonal signaling with metabolism in cell culture systems ^4^. The functions of mTOR are split between two multi-subunit complexes: mTOR complex 1 (mTORC1, consists mTOR, Raptor and Deptor, is sensitive to rapamycin) and mTOR complex 2 (mTORC2, contains mTOR, Rictor and Deptor, is insensitive to rapamycin) ^6,7^. Several studies determined the roles of each complex in adipose tissues, relying upon selective deletion of essential regulatory subunits by Cre-loxP recombination system ^8–12^. These studies shown that both complexes play important roles in lipid homeostasis, adipogenesis, glucose metabolism and insulin actions ^8–12^. However, most of the previous studies were achieved by deletion of either mTORC1 or mTORC2.

Concerning the efficiency and the specificity of different Cre strains (e.g., Fabp4-Cre, Myf5-Cre, Adipoq-Cre) ^13–15^, we used the more specific and efficient Adipoq-Cre to drive the deletion of loxP flanked genes in adipocytes ^16^. To directly and specifically delete both mTORC1 and mTORC2 in adipocytes, we generated adipocyte-specific mTOR knockout mice (Adipoq-mTOR) by crossing adipocyte-restricted Adiponectin-Cre (Adipoq-Cre) mice with mTOR^flox/flox^ mice ^16^. We demonstrated that adipocyte-specific deletion of mTOR decreased adipose tissue mass and induced browning of WAT, as well as caused insulin resistance and fatty liver ^16^. Furthermore, we reveled that ablation of mTOR inhibited the differentiation of pre-adipocytes through PPARγ signaling ^16^. Based on our results and the previous reports, the reduction of adipose tissue mass observed in Adipoq-mTOR mice might be due to the inhibited adipocyte differentiation and lipid accumulation caused by mTORC1 deletion ^9,17^. The insulin resistance in Adipoq-mTOR mice might mainly result from hepatic steatosis induced by deletion of mTORC2 in adipose tissues ^8,11^. Interestingly, either mTORC1 or mTORC2 deletion may promote white-to-brown switching ^8,18,19^. Thus, the browning of WAT in Adipoq-mTOR mice would be associated with mTORC1 and mTORC2 deficiency.

Lkb1 is a tumor suppressor and plays essential roles in various metabolic tissues. Using Fabp4-Cre, Zhang et al generated adipose tissue-specific Lkb1 knockout (Fabp4-Lkb1) mice and observed WAT mass reduction, postnatal growth retardation, and early death before weaning ^20^. These severe phenotypes of FABP4-Lkb1 mice suggest that Lkb1 regulates adipogenesis ^20^. Due to the efficiency and the leaky expression of Fabp4-Cre ^14,15^, we used Adipoq-Cre to drive Lkb1 deletion in WAT and BAT ^21^. Though the size and weight of various WAT depots were identical in the WT and Adipoq-Lkb1 mice, the BAT mass of Adipoq-Lkb1 mice was notably enlarged ^21^. Robust metabolic phenotypes were observed in Adipoq-Lkb1 mice, such as improved systemic insulin sensitivity, glucose tolerance and energy expenditure, and resistance to HFD-induced obesity ^21^. Molecular mechanisms studies revealed that ablation of Lkb1 upregulates the expression of BAT-specific genes and leads to expansion and browning of BAT through mTOR pathway ^21^. However, Adipoq-Cre is not perfect to conclusively distinguish the depot independent functions of a specific target, as it targets all mature adipocytes. Other Cre strains, such as Ucp1-Cre, should be taken into consideration for targeting BAT adipocyte.

Sufficient evidence suggested that Lkb1 is required for repression of mTOR under low ATP conditions in cultured cells ^22^. The defined intracellular mechanics of Lkb1-mTOR pathway, as well as the overlapping and distinct phenotypes observed in adipocyte-specific Lkb1 or mTOR single knockout mice prompted us to examine the complex interaction of Lkb1 and mTOR in *vivo*. Thus, we generated adipocyte-specific Lkb1 and mTOR DKO mice. DKO of Lkb1 and mTOR results in reductions of BAT and iWAT mass under normal chow diet or HFD ^16^, which is consistent with the decreased fat mass phenotype in adipocyte-specific mTORC1 single deletion (Adipoq-mTORC1) mice, but inconsistent with the expanded BAT phenotype in adipocyte-specific Lkb1 single deletion (Adipoq-Lkb1) mice ^16,21^. Notably, the expansion phenotype observed in the Lkb1 single deletion was overturned by mTORC1 ablation in the DKO mice ^9,21^, suggesting the key roles of mTORC1 in regulating adipose development. In addtion, DKO of Lkb1 and mTOR decreases the expression of the two key regulators of adipocyte differentiation and lipogenesis (PPARγ and C/EBPα) in adipose tissues ^16^. These findings suggest that Lkb1 regulates adipocyte differentiation and lipid accumulation mainly through mTOR pathway.

Several studies have highlighted that the strong association between Non-Alcoholic Fatty Liver Disease (NAFLD) and insulin resistance. Our previous work reported that Lkb1 single deficiency in adipose tissue protected mice from HFD-induced fatty liver associated with better glucose tolerance and higher insulin sensitivity ^21^. However, DKO mice developed fatty liver and displayed discordant glucose tolerance (enhanced) and insulin sensitivity (reduced) under HFD ^5^. Likewise, Adipoq-Cre mediated mTORC1 deletion mice developed systemic metabolic disease including hepatomegaly, hepatic steatosis and insulin intolerance ^8,9^. mTORC2 knockout mice had insulin resistance and mild hepatic steatosis ^12^. Adipocyte-specific deletion of mTOR also caused insulin resistance and increased hepatic steatosis ^16^. In addition, adipose-specific knockout of Rictor in mice led to mild glucose intolerance and defects in adipocyte glucose uptake ^12^. The impaired insulin-mediated suppression of hepatic glucose production caused by mTORC2 might be account for profound fatty liver or dysplastic hepatic nodules in DKO mice.

We shown that DKO of Lkb1 and mTOR did not alter whole-body energy metabolism but caused downregulation of UCP1 and mitochondrial related genes ^16^. Previous studies also reported that UCP1 expression in WAT induced by cold or βAR3 agonists was regulated by functional mTORC1 that activation enhances mitochondrial activity ^23-25^. Consistently, the BAT from Adipoq-Raptor mice failed to adaptation to cold ^18^. Though mTORC2 can sustains thermogenesis via glucose uptake and glycolysis in brown adipose tissue, it is not required for cold-induced lipid droplet mobilization, mitochondrial uncoupling, or β-oxidation ^26^. In our previous publication, we revealed that Lkb1 could control brown adipose tissue growth and thermogenesis through AMPK-mTOR pathway ^21^. Taken together, the deficiency of mTOR overturned the positive effect of Lkb1 deletion on thermogenesis and mitochondrial function in the DKO mice.

The Lkb1-KO, mTOR-KO, mTORC1-KO, mTORC2-KO and Lkb1/mTOR DKO mice all display resistant to HFD-induced obesity ^4,16,21^. However, the protection mechanism of HFD-induced obesity in these models is different. In Lkb1 single KO Adipoq-Lkb1 mice, the improved systemic insulin sensitivity and energy expenditure result in resistance to HFD-induced obesity ^21^. Differently, the inhibited adipocyte differentiation and lipid accumulation induced by mTORC1 may explain the resistance to HFD-induced obesity in mTORC1, mTOR KO and LKB1/mTOR DKO mice ^4,5,16^. In addition, mTORC2 KO mice anti-obesity through activation of thermogenesis in BAT ^26^.

Additionally, the Adipoq-Lkb1 KO mice develop hindlimb paralysis at mid-age ^27^. As the Adipoq-Lkb1 KO mice have better metabolism ^21^, the metabolism alteration could not explain the effect of BAT on peripheral nerve lesion. Interestingly, they shown that inflammatory cytokines produced by Lkb1-KO BAT lead to infiltration of macrophages into the sciatic nerve and axon degeneration, and subsequently cause hindlimb paralysis ^27^. Indeed, deletion of Lkb1 in adipose tissues activates mTOR signaling ^21^, which plays a pivotal role in regulating immune responses ^28^. Application of rapamycin (mTOR inhibitor) could rescue the hindlimb paralysis in Adipoq-Lkb1 KO mice ^27^. Consistently, DKO mice were behaviorally normal and completely free of hindlimb paralysis through the age of 2-year-old ^27^. This phenotype was not reported in other transgenic models, the mTOR-KO ^21^, the mTORC1-KO ^9^, and the mTORC2-KO ^12^ mice. These results suggest that Lkb1 deletion in BAT results in cross-talk between BAT and other tissues.

The similarities and differences among the DKO ^5^, the Lkb1-KO ^21^, the mTOR-KO ^16^, the mTORC1-KO ^9^, and the mTORC2-KO ^12^ mice were presented in Figure 1. Taken together, our study suggest that Lkb1/mTOR DKO in adipocytes results in overlapping and distinct metabolic phenotypes, and mTOR KO largely overrides the effect of Lkb1 KO, as mTOR acts as a downstream target gene of Lkb1 ^5^. The completion of this study directly revealed the effects of double knockout of Lkb1 and mTOR on adipose growth, energy expenditure, glucose metabolism, insulin sensitivity and inflammatory responses. Our results combined with other reports could provide novel insights into understanding the roles of Lkb1 and mTOR in regulating adipose development and energy metabolism, as well as crosstalk between BAT and other tissues. And these kinds of studies are useful for determining potential therapeutically targets and developing strategies to combat obesity and other metabolic diseases.

**Figure 1.**
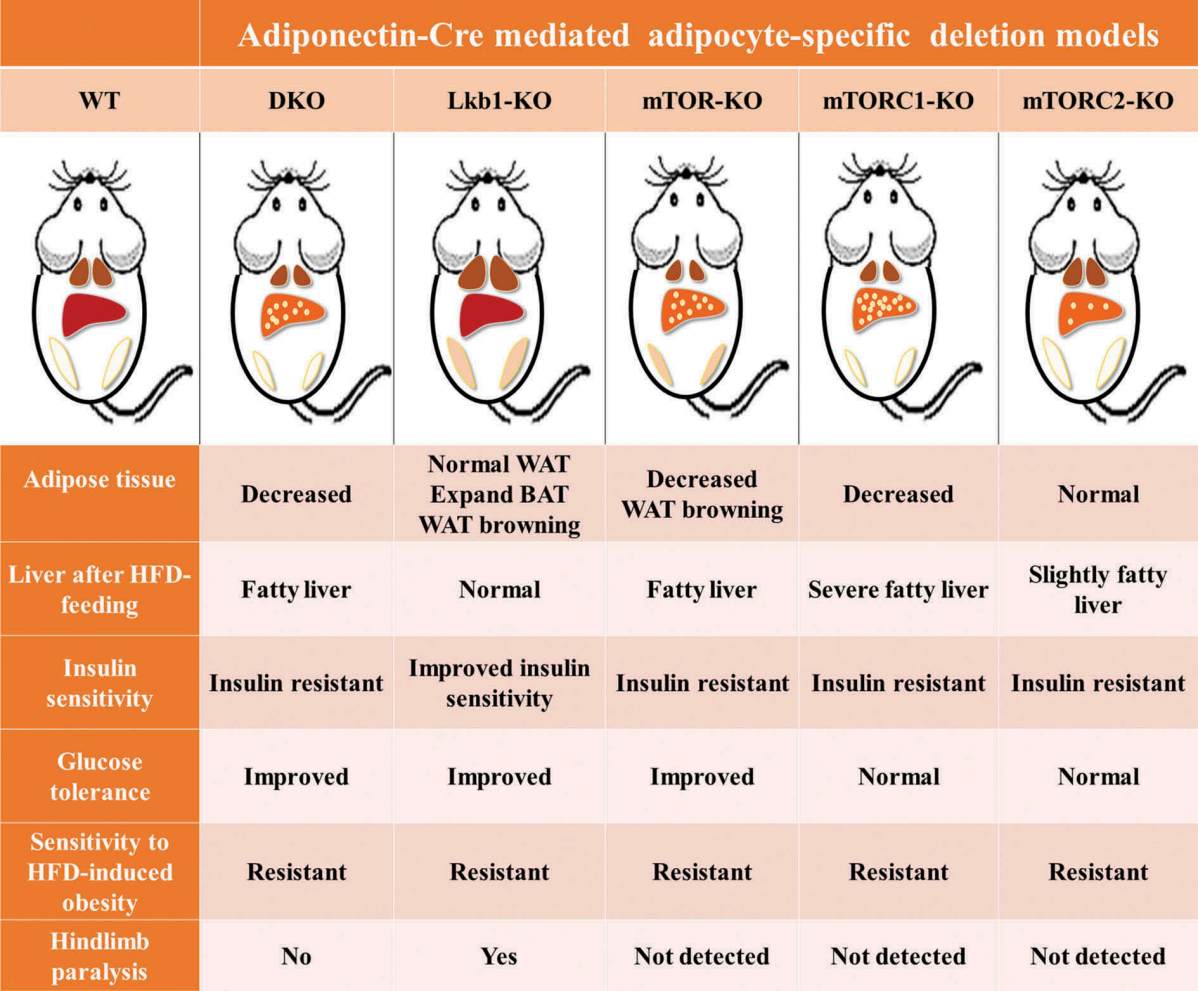
Adipocyte-specific Lkb1/mTOR, or Lkb1, or mTOR, or mTORC1 (Raptor) or mTORC2 (Rictor) ablation causes severe but different metabolic complications.
